# Soluble form of CTLA‐4 is a good predictor for tumor recurrence after radiofrequency ablation in hepatocellular carcinoma patients

**DOI:** 10.1002/cam4.4760

**Published:** 2022-04-18

**Authors:** Wei Teng, Wen‐Juei Jeng, Wei‐Ting Chen, Chen‐Chun Lin, Chun‐Yen Lin, Shi‐Ming Lin, I‐Shyan Sheen

**Affiliations:** ^1^ Department of Gastroenterology and Hepatology, Chang Gung Memorial Hospital Linkou Medical Center TaoYuan Taiwan; ^2^ Institute of Clinical Medicine National Yang‐Ming Chiao‐Tung University Taipei Taiwan; ^3^ College of Medicine Chang Gung University Taoyuan Taiwan

**Keywords:** chronic hepatis C, hepatocellular carcinoma, prognosis, radiofrequency ablation, soluble form of cytotoxic‐T‐lymphocyte‐antigen‐4

## Abstract

**Background:**

A soluble form of cytotoxic‐T‐lymphocyte‐antigen‐4 (sCTLA‐4) is a prognostic biomarker for several cancers but remains unclear in HCC patients. The aim of study is to evaluate the predictive role of serum sCTLA‐4 levels for tumor recurrence of chronic hepatis C (CHC)‐HCC patients receiving radiofrequency ablation (RFA).

**Material and method:**

A prospective study recruiting 88 CHC‐HCC patients was done between 2013 and 2019. Cox regression analysis was used to determine the predictors of early recurrence. All tests were two‐tailed, and the level of statistical significance was set as *p* < 0.05.

**Results:**

During a median follow‐up of 44.4 months, 53 of the 88 (60.2%) CHC‐HCC patients encountered early recurrence within 2 years. The predictability of sCTLA‐4 for local recurrence (LR) and intrahepatic metastasis (IHM) by 2‐years using AUROC curve analysis were 0.740 and 0.715, respectively. Patients with high sCTLA‐4 levels (>9 ng/ml) encountered shorter recurrence‐free survival (RFS) for LR (log‐rank *p* = 0.017) but paradoxically longer RFS for IHM (log‐rank *p* = 0.007) compared to those with low levels (≤9 ng/ml). By multivariate Cox regression analysis, sCTLA‐4 levels and antiviral therapy were independent prognostic factor of early recurrence both in LR and IHM. A combination of baseline sCTLA‐4 and AFP level could improve the predictability of early LR and IHM with specificity of 80.0% and 79.7% and positive predictive value of 63.3% and 67.3%, respectively.

**Conclusions:**

sCTLA‐4 level is a good predictor for early HCC recurrence with higher levels indicating susceptibility to early LR, but protecting from early IHM.

## Graphical abstract

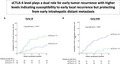

**Lay summary**

Baseline serum sCTLA‐4 is an independent predictor for early tumor recurrence in CHC‐HCC patients treated with curative RFA which plays a dual role, with higher levels indicating susceptibility to early LR, but protecting from early IHM.

## 1 INTRODUCTION

Hepatocellular carcinoma (HCC) ranks the third most common cause of cancer‐related deaths worldwide^1^ with viral hepatitis (e.g., HBV, HCV), alcoholic liver disease, and steatohepatitis being the major etiologies. Potentially curative treatment modalities for patients with hepatoma include radiofrequency ablation (RFA), resection, and transplantation.^2^, ^3^ However, overall prognosis is still unsatisfactory because of high recurrence rate like 5‐year recurrence rate is nearly 50% after resection^4^, ^5^, ^6^, ^7^ and 1‐year recurrence rate is close to 30% after RFA.^8^, ^9^, ^10^

The widen understanding of the relationship between the immune systems and tumor cells has paved the way for the use of immunotherapy tools in clinics in recent years. Cytotoxic T‐lymphocyte antigen 4 (CTLA‐4) plays an inhibitory role with a stronger binding affinity than CD28 to CD80 (B7‐1) and CD86 (B7‐2) on antigen‐presenting cells (APCs). Therefore, CTLA‐4 could block the interaction of B7 and CD28 and inhibit the activation and proliferation of T cells.^11^, ^12^ The importance of this CTLA‐4 molecule in antitumor immune responses has recently been highlighted by the success of anti‐CTLA‐4 monoclonal antibodies (mAbs), including ipilimumab, as cancer immunotherapy.^13^, ^14^

A soluble form CTLA‐4 (sCTLA‐4) which originates from alternative splicing results in the loss of a cysteine residue and is found in the serum as a soluble monomeric protein.^15^, ^16^ sCTLA‐4 also plays an important role in immune responses but with opposite effects. Unlike anti‐CTLA‐4 mAbs, which bind directly to the CTLA‐4 molecule, sCTLA‐4 competes with CD28 or membranous CTLA‐4 (mCTLA‐4) on the T cells for the B7 binding. Therefore, sCTLA‐4 may specifically inhibit T‐cell activation in the early stage by blocking the interaction of B7 and CD28.^17^ On the other hand, some studies also suggest sCTLA‐4 also contends for the binding of the inhibitory receptor mCTLA‐4 on the T cells with B7 on the APCs in effector T‐cell phase, causing a reduction of inhibitory signaling and maintaining effector T‐cell functionality.^18^, ^19^

The role of sCTLA‐4 in patients with solid tumors has also been investigated and likewise show contradictory results. These studies found that high levels of baseline sCTLA‐4 predicted longer recurrence‐free survival (RFS) in patients with malignant pleural mesothelioma^20^ while predicting shorter RFS in patients with glioma^21^ and colorectal cancers.^22^ However, it is still unclear the association between soluble CTLA‐4 and HCC patients. The aim of this study is therefore to analyze the value of baseline serum sCTLA‐4 level in predicting early tumor recurrence of HCC patients after complete RFA treatment.

## 2 PATIENTS AND METHODS

### 2.1 Patient selection and RFAprocedure

One‐hundred and ninety‐one HCC patients under RFA therapy between November 2013 and August 2019 were retrospectively recruited from Chang Gung Memorial Hospital, Linkou Medical Center. HCC is diagnosed by multiphasic, contrast‐enhanced imaging (CT or MRI scans) and/or pathology according to the European Association for the Study of the Liver/European Organization for Research and Treatment of Cancer (EASL/EORTC) diagnostic guidelines.^23^ Patients undergoing RFA for recurrent HCC and followed less than 2 years were excluded. The baseline levels of sCTLA‐4 were largely variable among different etiologies (Figure S1). In order to diminish the variability due to etiologies, we only enrolled chronic hepatitis C (CHC)‐HCC patients for further analysis.

Patients with Child‐Pugh class A or B liver profile, with three or less HCC nodules and the size of all the nodules ≤3 cm are indicated for RFA treatment.^23^ Patients were excluded if they encountered with refractory ascites, portal vein thrombosis, and extrahepatic metastasis.^24^ Percutaneous RFA was done by using straight electrodes under ultrasound guidance and artificial ascites or pleural effusion techniques^25^ were used when the target tumor was close to critical organs or in the difficult locations. All procedures were performed by three hepatologists with more than 15 years' experience in percutaneous thermal ablations.

Pre‐treatment liver function, alpha‐fetoprotein (AFP), platelet count, TNM stage, tumor size, age, gender, and serum sCTLA‐4 were measured within 3 days before RFA in all of the patients. In addition, follow‐up serum sCTLA‐4 within 3 days after RFA treatment were measured in 35 patients.

### 2.2 Study endpoints and follow‐up protocol

The study endpoint was recurrence‐free survival (RFS), defined as the time from curative HCC treatment to the first documented tumor recurrence by assessment of radiological images. Complete response was confirmed by validated image study (CT or MRI scans) of the abdomen approximately 1 month after treatment. The follow‐up protocol included clinical assessment by physical examination, biochemistry study as well as multiphasic CT or MRI every 3 months for the first 2 years, extended to 6 month intervals thereafter according to EASL/EORTC clinical practice guidelines.^23^ HCC recurrence was diagnosed based on image study confirming hyper‐vascularization in the arterial phase with washout in the portal venous or late venous phase. Early HCC recurrence was defined as^1^: the reappearance of the tumor located in the peripheral area of the treated site (local recurrence, LR) or^2^ tumors detected at distant sites from the primary tumor (intrahepatic distal metastasis, IHM) within 2 years.

### 2.3 Laboratory methods

Pre‐treatment blood cell counts, and biochemical tests were performed using automated techniques at the clinical pathology laboratories of the hospital. Whole blood with 8–10 ml was collected in the BD Vacutainer Serum Plus Blood Collection Tube. After collection of the whole blood, we leave it undisturbed at room temperature to allow the blood to clot and then remove the clot by centrifuging at 1500 *g* for 15 min in a refrigerated centrifuge. Following centrifugation, the supernatants were immediately transferred to the new 5 ml polypropylene tube, and store the sample in the −80°C fridge. The soluble form of CTLA‐4 was measured by using a solid phase sandwich enzyme‐linked immunosorbent serologic assay (ELISA) kit (minimum detectable level is 0.5 ng/ml). Serum hepatitis markers, including anti‐hepatitis C virus, were assayed using the Enzyme Immunoassay (EIA) kit (Abbott Diagnostics, North Chicago, IL).

### 2.4 Statistical analysis

Categorical variables between the two groups were calculated by Chi‐square. Descriptive data with normal distribution are presented as mean ± standard deviation (SD), otherwise as median (interquartile range, IQR). The independent Student's test and Mann–Whitney *U* test were used to assess differences between groups in normal distributed and non‐normal distributed groups separately. Stepwise Cox regression models were used to determine the predictors of early tumor recurrence. A two‐tailed *p* value <0.05 was considered as statistically significant. Area under the receiver operating characteristic (AUROC) and Youden Index were applied for optimal cutoff value of sCTLA4 for prediction of tumor recurrence. Statistical analysis was done with SPSS software, version 20.0 (IBM Corp., Armonk, NY, USA).

## 3 RESULTS

### 3.1 Clinical baseline Characteristics and post RFAclinical outcomes

The clinical characteristics of the 88 participants are shown in Table 1. Median age was 67.2‐year‐old and 47 (53.4%) were male. Most patients were Child‐Turcotte‐Pugh (CTP) class A (*n* = 77, 87.5%) and TNM stage I (*n* = 61, 69.3%). Twenty‐eight (31.8%) patients had achieved sustained virologic response (SVR) before HCC diagnosis and study enrollment as well as most were receiving peginterferon‐based therapy (*n* = 22, 78.6%). Fifty‐three (60.2%) patients encountered early tumor recurrence within 2 years during the median follow‐up duration with 44.4 (range 24.9–82.4) months. The most frequent type of recurrence was IHM (54.7%) with median RFS 12.9 months followed LR (45.3%) with median RFS 8.3 months. Among these patients, 37 patients received RFA and 16 patients received TACE after tumor recurrence.

**TABLE 1:** Baseline characteristics of enrolled patients

Variable	Overall (*N* = 88)
Age (years)	67.2 (IQR 61.8–75.0)
Gender (male, %)	47 (53.4)
TNM stage I/II, *n* (%)	61/27 (69.3/30.7)
Antiviral therapy, *n* (%)	28 (31.8)
CTP class A/B, *n* (%)	77/11 (87.5/12.5)
NLR	2.0 ± 1.2
Total bilirubin (mg/dl)	0.7 (IQR 0.5–1.2)
ALT (U/L)	38 (IQR 25–67)
Albumin (g/dl)	3.80 (IQR 3.39–4.16)
AFP (ng/ml)	17 (IQR 5–80)
Platelet (10^3^/μl)	116 (IQR 74–163)
sCTLA‐4 (ng/ml)	9.8 ± 10.4
Target lesion size (cm)	2.0 (IQR 1.5–2.7)
Tumor number 1/2, *n* (%)	61/27 (69.3/30.7)
Early recurrence within 2 years, *n* (%)	53 (60.2)
LR, *n* (%)	24 (45.3)
IHM, *n* (%)	29 (54.7)
EHM, *n* (%)	12 (13.6)
Mortality, *n* (%)	23 (26.1)
Follow‐up duration (months)	44.4 (range 24.9–82.4)

Abbreviations: AFP, alpha‐fetoprotein; ALT, alanine aminotransferase; CTP, Child‐Turcotte‐Pugh; EHM, extrahepatic metastasis; HCC, hepatocellular carcinoma; IHM, intrahepatic distant metastasis; LR, local recurrence; NLR, neutrophil‐to‐lymphocyte ratio; sCTLA‐4, soluble form of cytotoxic‐T‐lymphocyte‐antigen‐4.

### 3.2 Association of serum sCTLA‐4 levels with clinical parameters in CHC‐HCCpatients

The correlations between baseline serum sCTLA‐4 and the clinical parameters in 88 CHC‐HCC patients are analyzed (Table S1). The results demonstrated that sCTLA‐4 level was positively associated with the age, Child‐Pugh (CTP) class, TNM stage, tumor size, and neutrophil‐to‐lymphocyte ratio (NLR). There were no significant association between serum sCTLA‐4 levels and other clinical parameters such as gender, antiviral therapy, liver function test, and AFP level (all *p* value >0.05).

### 3.3 The correlation between sCTLA‐4 and early HCCrecurrence

**FIGURE 1:**
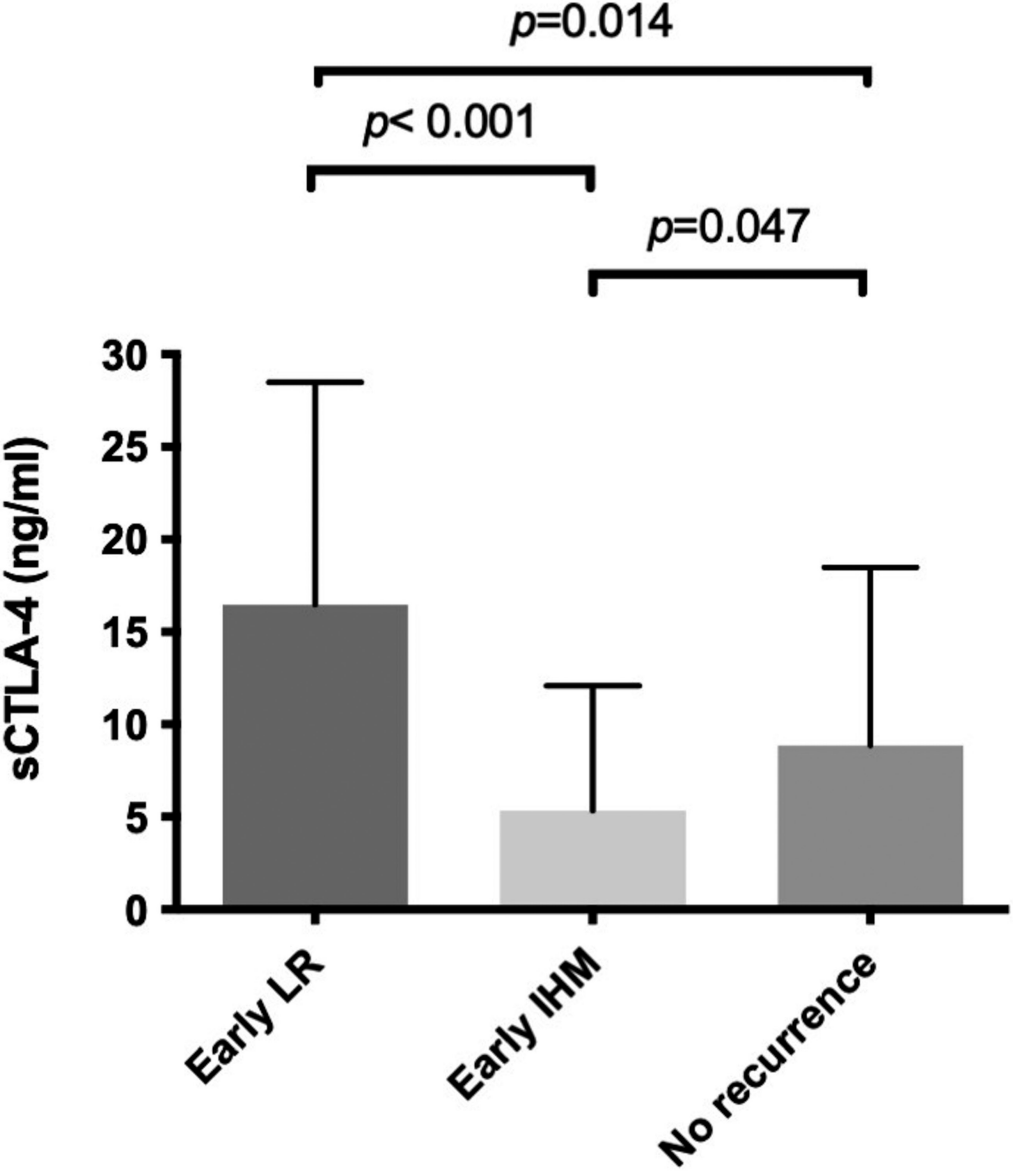
A serum sCTLA‐4 is differently found in the patients with recurrence pattern and non‐recurrence. Abbreviation: IHM, intrahepatic distant metastasis; LR, local tumor recurrence; sCTLA‐4, soluble form of cytotoxic‐T‐lymphocyte‐antigen‐4

**FIGURE 2:**
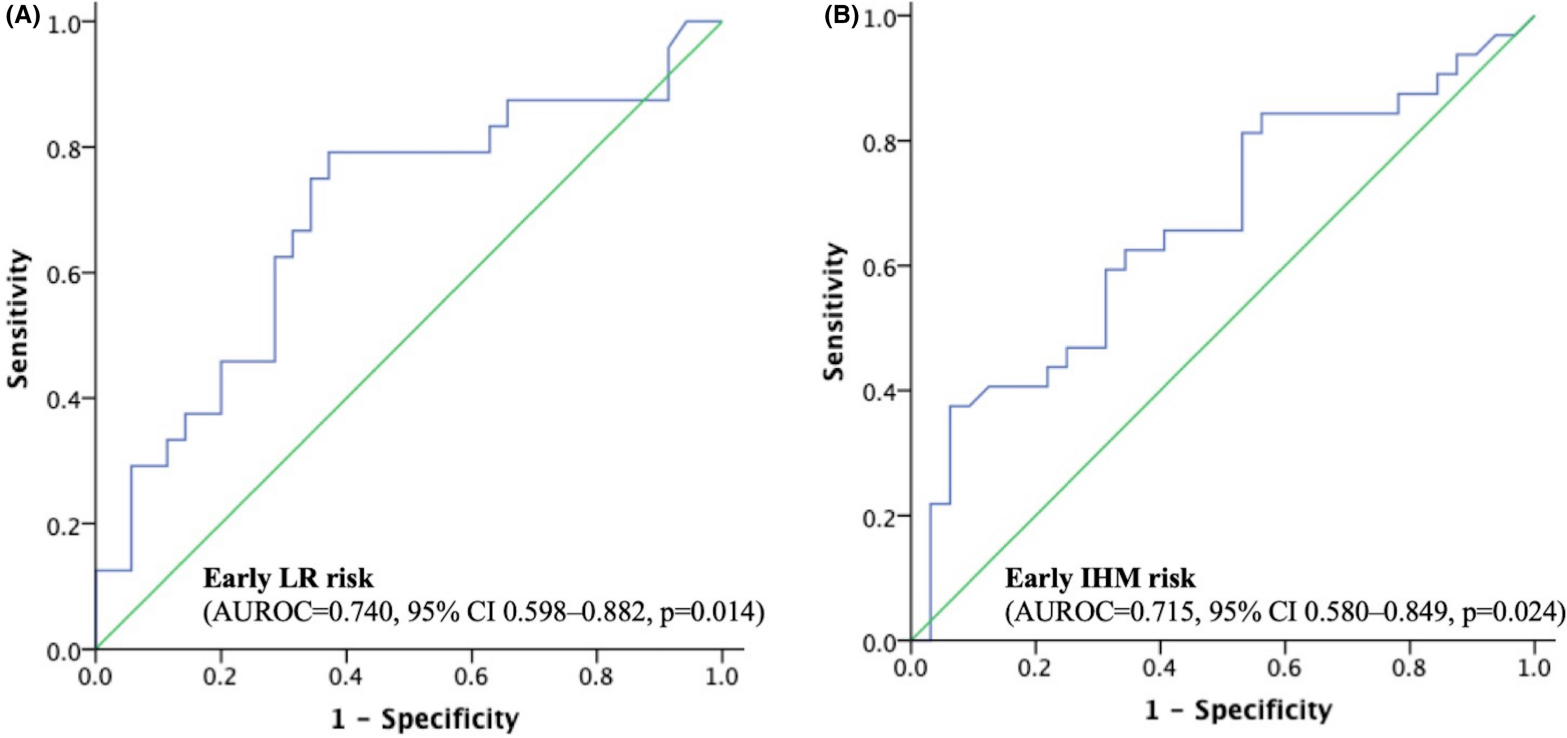
ROC curves for risk of HCC recurrence prediction. (A) Early local recurrence. (B) Early intrahepatic distant metastasis

The differences of baseline features among patients with and without early recurrence are shown in Table S2. When compared with non‐early recurrence group, patients encountering early recurrence had a greater likelihood of TNM stage II (37.7% vs. 20.0%, *p* = 0.047) and less antiviral therapy (17.0% vs. 54.3%, *p* < 0.001). The pre‐treatment mean sCTLA‐4 levels between patients with and without early recurrence were comparable (10.4 ng/ml vs. 8.7 ng/ml, *p* = 0.520). When recurrence patterns were further stratified into IHM and LR, the differences of baseline features among patients are shown in Table 2. sCTLA‐4 levels in patients without tumor recurrence were significantly lower than in those with early LR (8.7 vs. 16.5 ng/ml, *p* = 0.014) but higher than in subjects with IHM (8.7 vs. 5.3 ng/ml, *p* = 0.047) (Figure 1). To determine the best cutoff value of sCTLA‐4 for prediction of LR and IHM, AUROC curve and Youden index analysis were performed. A cutoff of sCTLA‐4 of 9 ng/ml was associated with AUROC of 0.740 [95% confidence interval (CI): 0.598–0.882, *p* = 0.014] and 0.715 (95% CI: 0.580–0.849, *p* = 0.024) for predicting both LR and IHM, respectively (Figure 2).

**TABLE 2:** Comparisons of baseline features of patients with and without tumor recurrence

Variables	Early tumor recurrence			*p* value
	No (*N* = 35)	LR (*N* = 24)	IHM (*N* = 29)	
Age (years)	66.9 (IQR 61.1–75.0)	67.2 (IQR 60.6–75.0)	68.2 (IQR 62.1–75.3)	0.843
Gender (male, %)	16 (45.7)	14 (58.3)	17 (58.6)	0.501
TNM stage I/II, *n* (%)	28/7 (80.0/20.0)	17/7 (70.8/29.2)	16/13 (55.2/44.8)	0.099
Antiviral therapy, *n* (%)	19 (54.3)	5 (20.8)	4 (13.8)	0.001
CTP class A/B, *n* (%)	32/3 (91.4/8.6)	20/4 (83.3/16.7)	25/4 (86.2/13.8)	0.632
NLR	1.7 ± 0.9	2.3 ± 1.6	1.9 ± 1.2	0.234
Total bilirubin (mg/dl)	0.7 (IQR 0.5–1.0)	1.1 (IQR 0.7–1.5)	0.7 (IQR 0.5–1.0)	0.006
ALT (U/L)	42 (IQR 25–69)	42 (IQR 25–72)	35 (IQR 25–64)	0.798
Albumin (g/dl)	3.86 (IQR 3.56–4.21)	3.94 (IQR 3.27–4.14)	3.57 (IQR 3.37–4.05)	0.597
AFP (ng/ml)	17 (IQR 5–66)	13 (IQR 5–100)	26 (IQR 5–127)	0.602
Platelet (× 10^3^/μl)	122 (IQR 85–164)	97 (IQR 59–155)	120 (IQR 64–172)	0.461
sCTLA‐4 (ng/ml)	8.7 ± 9.6	16.5 ± 12.0	5.3 ± 6.7	<0.001
Target lesion size (cm)	1.7 (IQR 1.4–2.6)	2.3 (IQR 1.7–3.2)	1.9 (IQR 1.5–2.5)	0.197
Tumor number 1/2, *n* (%)	28/7 (80.0/20.0)	17/7 (70.8/29.2)	16/13 (55.2/44.8)	0.099
Mortality, *n* (%)	7 (20.0)	6 (25.0)	10 (34.5)	0.418
Follow‐up duration (months)	52.1 (IQR 32.0–63.6)	42.1 (IQR 29.2–49.7)	42.0 (IQR 20.5–56.3)	0.292

Abbreviations: AFP, alpha‐fetoprotein; ALT, alanine aminotransferase; CTP, Child‐Turcotte‐Pugh; HCC, hepatocellular carcinoma; IHM, intrahepatic distant metastasis; LR, local recurrence; NLR, neutrophil‐to‐lymphocyte ratio; sCTLA‐4, soluble form of cytotoxic‐T‐lymphocyte‐antigen‐4.

In a subgroup of 35 patients who had prospective sequential blood sampling within 3 days after RFA treatment, the mean sCTLA‐4 level was elevated after RFA (pre‐RFA vs. post RFA: 13.8 vs. 16.8 ng/ml, *p* = 0.040) (Figure S2A). For further analysis, the phenomenon of elevation of sCTLA‐4 after RFA only found in patients with early recurrence (*p* = 0.038) but not in patients without early recurrence (*p* = 0.817) (Figure S2B).

### 3.4 Predictors of early tumor recurrence after RFAtreatment

By univariate Cox regression analysis, the use of antiviral therapy, AFP > 10 ng/ml and sCTLA‐4 > 9 ng/ml were predictors of early LR (Table 3) while baseline TNM stage, use of antiviral therapy, AFP > 10 ng/ml and sCTLA‐4 > 9 ng/ml were predictors of early IHM (Table 4). By multivariate Cox regression analysis, antiviral therapy was a protective factor both for LR [adjusted HR: 0.363 (95% CI: 0.135–0.979), *p* = 0.045] (Table 3) and IHM [adjusted HR: 0.256 (95% CI: 0.085–0.773), *p* = 0.016] (Table 4). However, the paradoxical predictive value of sCTLA‐4 >9 ng/ml was observed that increased susceptibility of LR [adjusted HR:2.434 (95% CI: 1.030–5.751), *p* = 0.043] while being a protective factor for IHM [adjusted HR:0.190 (95% CI: 0.045–0.809), *p* = 0.025].

**TABLE 3:** Cox's proportional hazards model for predictors of early local recurrence

Variables	Univariate			Multivariate		
	HR	95% CI	*p* value	HR	95% CI	*p* value
Age >65 y/o (vs. ≤65 y/o)	0.978	0.434–2.205	0.958			
Male (vs. female)	1.439	0.638–3.246	0.380			
TNM stage II (vs. I)	1.335	0.553–3.224	0.521			
Antiviral therapy (vs. no)	0.320	0.119–0.859	0.024	0.363	0.135–0.979	0.045
CTP class B (vs. A)	2.401	0.814–7.081	0.112			
APRI >2 (vs. ≤2)	1.099	0.470–2.570	0.827			
FIB‐4 >3.25 (vs. ≤3.25)	1.831	0.758–4.423	0.179			
ALBI grade II/III (vs. I)	1.236	0.549–2.784	0.609			
NLR >2 (vs. ≤2)	1.087	0.476–2.481	0.843			
AFP >10 (vs. ≤10 ng/ml)	1.192	1.029–2.686	0.042	1.171	0.516–2.656	0.706
sCTLA‐4 >9 (vs. ≤ 9 ng/ml)	2.707	1.157–6.334	0.022	2.434	1.030–5.751	0.043
Tumor size >3 cm (vs. ≤3 cm)	1.500	0.621–3.626	0.368			
Tumor number 2 (vs. 1)	1.335	0.553–3.224	0.521			

Abbreviations: AFP, alpha‐fetoprotein; ALBI, albumin‐bilirubin; APRI, AST to Platelet Ratio Index; CTP, Child‐Turcotte‐Pugh; HCC hepatocellular carcinoma; NLR, neutrophil‐to‐lymphocyte ratio; sCTLA‐4, soluble form of cytotoxic‐T‐lymphocyte‐antigen‐4

**TABLE 4:** Cox's proportional hazards model for predictors of early intrahepatic metastasis

Variables	Univariate			Multivariate		
	HR	95% CI	*p* value	HR	95% CI	*p* value
Age >65 y/o (vs. ≤65 y/o)	1.122	0.521–2.417	0.769			
Male (vs. female)	1.428	0.681–2.993	0.346			
TNM stage II/III (vs. I)	2.166	1.040–4.513	0.039	1.788	0.843–3.794	0.130
Anti‐viral therapy (vs. no)	0.207	0.072–0.596	0.004	0.256	0.085–0.773	0.016
CTP class B (vs. A)	1.850	0.641–5.341	0.256			
APRI >2 (vs. ≤2)	1.288	0.608–2.727	0.509			
FIB‐4 >3.25 (vs. ≤3.25)	1.705	0.774–3.757	0.185			
ALBI grade II (vs. I)	1.167	0.557–2.445	0.682			
NLR >2 (vs. ≤2)	1.299	0.587–2.872	0.519			
AFP >10 (vs. ≤ 10 ng/ml)	1.565	1.027–3.370	0.033	1.043	0.472–2.302	0.617
sCTLA‐4 >9 (vs. ≤9 ng/ml)	0.175	0.042–0.740	0.018	0.190	0.045–0.809	0.025
Tumor size >3 cm (vs. ≤3 cm)	1.258	0.433–3.654	0.673			
Tumor number 2 (vs. 1)	2.166	0.940–4.513	0.059			

Abbreviations: AFP, alpha‐fetoprotein; ALBI, Albumin‐bilirubin; APRI, AST to Platelet Ratio Index; CTP, Child‐Turcotte‐Pugh; HCC hepatocellular carcinoma; NLR, neutrophil‐to‐lymphocyte ratio; sCTLA‐4, soluble form of cytotoxic‐T‐lymphocyte‐antigen‐4.

In the patients with high level of sCTLA‐4 (>9 ng/ml), 16 (53.3%) patients encountered early LR and only 2 (6.7%) had early IHM. The median time to early LR and IHM in patients with baseline sCTLA‐4 >9 and ≤9 ng/ml were 8.1, 16.5 vs. 11.5, 8.5 months, respectively. Comparing the predictive value for early LR between sCTLA‐4 and AFP, sCTLA‐4 had better AUROC than AFP (0.740 vs. 0.541, *p* = 0.037), with sensitivity, specificity, PPV, and NPV of 76.7%, 74.2%, 57.1%, and 84.2% by sCTLA‐4 > 9 ng/ml and 68.3%, 61.5%, 52.4%, and 55.7% by AFP >10 ng/ml. When regarding to early IHM, sCTLA‐4 still had a better AUROC than AFP (0.715 vs. 0.558, *p* = 0.020) with sensitivity, specificity, PPV, and NPV of 93.1%, 65.7%, 54.0%, and 85.7% by sCTLA‐4 ≤ 9 ng/ml and 65.5%, 61.5%, 50.0%, and 65.5% by AFP >10 ng/ml. Furthermore, evaluation of the possibility of early LR and IHM in combination with AFP of 10 ng/ml and sCTLA‐4 of 9 ng/ml improved specificity to 80.0% and 79.7% as well as positive predictive value to 63.3% and 67.3% compared to either sCTLA‐4 or AFP alone, respectively (Table 5).

**TABLE 5:** Accuracy for prediction of early hepatocellular carcinoma recurrence using cutoff point of sCTLA‐4 and AFP

	Early LR			Early IHM		
	sCTLA‐4 >9 ng/ml	AFP >10 ng/ml	sCTLA‐4 >9 ng/ml & AFP >10 ng/ml	sCTLA‐4 ≤9 ng/ml	AFP >10 ng/ml	sCTLA‐4 ≤9 ng/ml & AFP >10 ng/ml
Sensitivity	76.7%	68.3%	73.3%	93.1%	65.5%	75.5%
Specificity	74.2%	61.5%	80.0%	65.7%	61.5%	79.7%
PPV	57.1%	52.4%	63.3%	54.0%	50.0%	67.3%
NPV	84.2%	55.7%	73.6%	85.7%	65.5%	79.7%

Abbreviations: AFP, alpha‐fetoprotein; IHM, intrahepatic metastasis; LR, local recurrence; NPV, negative predictive value; PPV, positive predictive value; sCTLA‐4, soluble form of cytotoxic‐T‐lymphocyte‐antigen‐4.

### 3.5 The predictive value of serum sCTLA‐4 in predicting tumor recurrence

**FIGURE 3:**
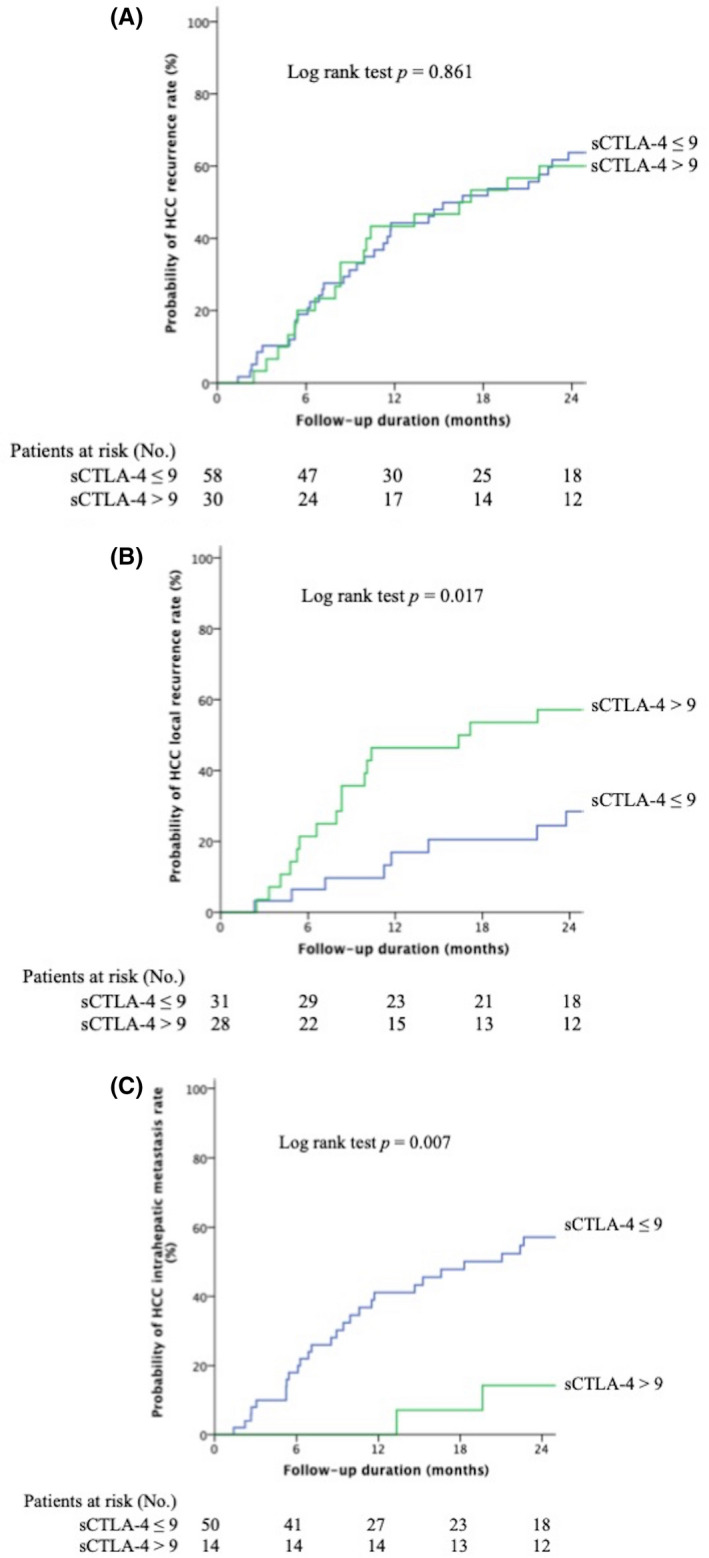
Kaplan–Meier curves showing the cumulative recurrence rate of patients with high and low baseline sCTLA‐4. (A) No significant difference between overall early recurrence. (B) Patients with higher baseline sCTLA‐4 (>9 ng/ml) are prone to have early LR. (C) In the contrary, patients with low baseline sCTLA‐4 (≤9 ng/ml) are prone to have early IHM. Abbreviation: LR, local tumor recurrence; IHM, intrahepatic distant metastasis; sCTLA‐4, soluble form of cytotoxic‐T‐lymphocyte‐antigen‐4

If taking overall early recurrence into account, there was no significant difference in cumulative early recurrence rate between the patients with high or low baseline level of sCTLA‐4 (log‐rank *p* = 0.861, Figure 3A). However, patients with baseline sCTLA‐4 >9 ng/ml had a higher 2‐year cumulative LR rate (log‐rank *p* = 0.017) (Figure 3B) but a lower 2‐year cumulative IHM rate (log‐rank *p* = 0.007) (Figure 3C). If combining sCTLA‐4 and AFP, the highest risk group of 2‐year cumulative incidence rate of LR was up to 56% when sCTLA‐4 >9 ng/ml and AFP >10 ng/ml (Figure S3) while 2‐year cumulative incidence rate of IHM was up to 64% when sCTLA‐4 ≤9 ng/ml concomitant with AFP >10 ng/ml (Figure S4).

## 4 DISCUSSION

RFA is a curative treatment for small HCC not suitable for resection or transplantation. However, long‐term survival is unsatisfactory because of high recurrence rate. Serum AFP is frequently used not only for diagnosis, but also as a predictor of treatment response in clinical practice because it is thought to consistently reflect tumor burden and activity.^26^, ^27^ However, nearly 30% HCC patients have normal AFP levels at the time of diagnosis and these levels usually remain low even at advanced stages.^28^ Therefore, identification of new biomarkers with higher sensitivity as well as specificity in predicting prognosis of HCC is therefore urgent. To our knowledge, this is the first study to determine that baseline sCTLA‐4 level can be an important predictor for HCC recurrence in CHC‐HCC patients. Interestingly, sCTLA‐4 plays a dual role, with higher levels indicating susceptibility to early LR, but protecting from early IHM. Baseline sCTLA‐4 level had better sensitivity, specificity, PPV, and NPV than AFP for predicting of early tumor recurrence. Furthermore, a combination of AFP and sCTLA‐4 improved specificity and PPV better than using either AFP or sCTLA‐4 alone.

Looking at the wider context, tumor immunology is an increasingly significant issue and immune checkpoints are particularly important within this field. CTLA‐4 on the surface of T cells is widely considered to be a critical immunomodulatory molecule in controlling T‐cell‐mediated immune responses and suppressing antitumor effects.^29^, ^30^ Soluble CTLA‐4 is derived from an alternatively spliced isoform of full‐length CTLA‐4 and secreted by several cell types including regulatory T cells (Treg),^31^ monocytes,^32^ melanoma cell lines,^33^ and pituitary gland cells.^34^ sCTLA‐4 is capable of binding to B7.1/B7.2 then blocking the B7/CD28 signaling pathway for T‐cell activation by the MYPPPY motif located in the extracellular domain of sCTLA‐4,^35^ leading to immunosuppressive function.^36^ Supporting this, several studies have shown that high serum sCTLA‐4 levels predicted shorter RFS in patients with glioma^21^ and colorectal cancer.^22^ As for the hepatocellular carcinoma, Kozuca et al.^37^ tried to elucidate the associations of 16 soluble immune checkpoint proteins including sCTLA‐4 with HCC development during nucleos(t)ide analogue (NA) treatment in 122 chronic hepatitis B (CHB) patients, However, there was no significant correlation between sCTLA‐4 and HCC development. Nevertheless, when consider the tumor recurrence, we have now shown in this study serum sCTLA‐4 level is associated with tumor recurrence in CHC‐HCC patients with high sCTLA‐4 levels had shorter RFS for early LR.

However, sCTLS‐4 is also a molecule with dual roles in immune responses.^36^ sCTLA‐4 could also compete with mCTLA‐4 on the effector T cells for the binding with B7.1/B7.2 on the APCs, reducing the inhibitory signaling in the effector T cells and maintaining the functionality of effector T cells. Based on this, higher serum sCTLA‐4 level could protect patients from cancer recurrence by preventing inhibitory signaling in effector T cells. Indeed, some reports have demonstrated that higher baseline levels of sCTLA‐4 predicted longer RFS in malignant pleural mesothelioma.^20^ Similarly, we also found that high sCTLA‐4 level is significantly associated with reduced incidence of IHM.

The question of how sCTLA‐4 levels significantly predict LR and IHM but in opposing directions is an intriguing but unresolved puzzle. However, LR and IHM are two different types of recurrence after loco‐regional treatment. It is therefore possible to speculate that LR could represent the regrowth of the residual tumor cells from the original tumor.^38^ Thus, it is possible that a higher sCTLA‐4 level inhibits the activation of potent antitumor T cells by blocking B7/CD28 interactions, which in turn helps the residual tumor to grow and increases the incidence of local recurrence. On the other hand, IHM may represent a new growth of tumor cells from de novo carcinogenesis of cirrhotic liver rather than from the primary tumor.^39^ Consequently, a higher level of sCTLA‐4 could reduce the inhibition of effector T cells by blocking B7/CTLA‐4 interaction, thereby decreasing de novo carcinogenesis and IHM incidence. It is not surprising that immediate elevation of sCTLA‐4 level within 3 days after ablation therapy which may reflect acute inflammatory status. Liu et al^40^ also found that expression of sCTLA‐4 within 3 days after therapy including radiotherapy and chemotherapy higher than that of 1 day before revealed good prognosis among patients with multiple kinds of cancer. The predictive value of outcome in which time‐point post‐therapy still need to be further investigated.

As a further point, it is well‐known that virus clearance is highly related to reduction of tumor recurrence in CHC‐HCC patients after RFA whether treated by IFN‐based^41^ or DAA‐based therapy.^42^ In the current study, most patients received IFN‐based therapy indeed decrease 2‐year tumor recurrence rate compared with untreated group (32.1 vs. 73.3%, *p* < 0.001). IFN‐α exerts potent antiviral activity via stimulation of IFN‐stimulated genes (ISGs) and its downstream signaling pathway^43^ which causes T‐cell homeostasis after SVR^44^ and immune modulation effect contributing to HCC reduction. The analyses of sCTLA‐4 and clinical parameters confirmed that sCTLA‐4 level were negatively correlated with antiviral therapy, although this did not reach statistical significance in the current study.

This study still had some limitations. First, the number of patients were too small, and the follow‐up period was relatively short for drawing definitive conclusions. Consequently, this study was focused on early recurrence (within 2 years) and the role of sCTLA‐4 in late recurrence (more than 2 years) remains to be investigated. Second, the patient cohort did not include chronic hepatitis C patients without tumor as a control group. Third, we have not conducted mechanistic studies to determine the functional impact of soluble immune checkpoint‐related proteins. Finally, our results should be interpreted carefully when considering populations beyond CHC patients.

In conclusion, baseline serum sCTLA‐4 is an independent predictor for early tumor recurrence in CHC‐HCC patients treated with curative RFA. Although the discrepant roles of sCTLA‐4 in determining different recurrence pattern, it may serve as a practical guidance to determine the follow‐up principle after the ablation. To lower the incidence rate of LR, early CT/MRI scan within 7 days after RFA might be necessary to check ablative safety margins^45^ in patients with higher baseline sCTLA‐4 level. Otherwise, regular image study should be performed every 3 months in the first 2 years to lower recurrence rate followed EASL/EORTC clinical practice guidelines.^23^ Further investigation with large population is indicated to validate the association between sCTLA‐4 level and the outcome of HCC treatment, as well as to establish the underlying mechanisms.

## CONFLICT OF INTEREST

The authors declare no potential conflict of interest.

## AUTHOR CONTRIBUTIONS

Wei Teng: data acquisition, statistical analysis, draft writing; Wen‐Juei Jeng: study concept, design, and manuscript revision; Wei‐Ting Chen: data acquisition; Chen‐Chun Lin: data acquisition; Chun‐Yen Lin: study concept, design and supervision, and manuscript revision; Shi‐Ming Lin: study concept; I‐Shyan Sheen: study concept, design, and statistical analysis.

## ETHICS APPROVAL STATEMENT

This study gained consent to the Institutional Review Board of Linkou Chang Gung Memorial Hospital committee (IRB number: 201602043B0) and was conducted ethically based on the World Medical Association Declaration of Helsinki. All the enrolled patients had signed the written informed consent.

## Supporting information


Figure S1

Figure S2

Figure S3

Figure S4

Table S1

Table S2

## Data Availability

N/A
