# Supplementary figures and images for: Soluble form of CTLA‐4 is a good predictor for tumor recurrence after radiofrequency ablation in hepatocellular carcinoma patients
Source: Cancer Med. 2022 Apr 18;11(20):3786–95. doi: 10.1002/cam4.4760 (PMC9582685; doi:10.1002/cam4.4760)

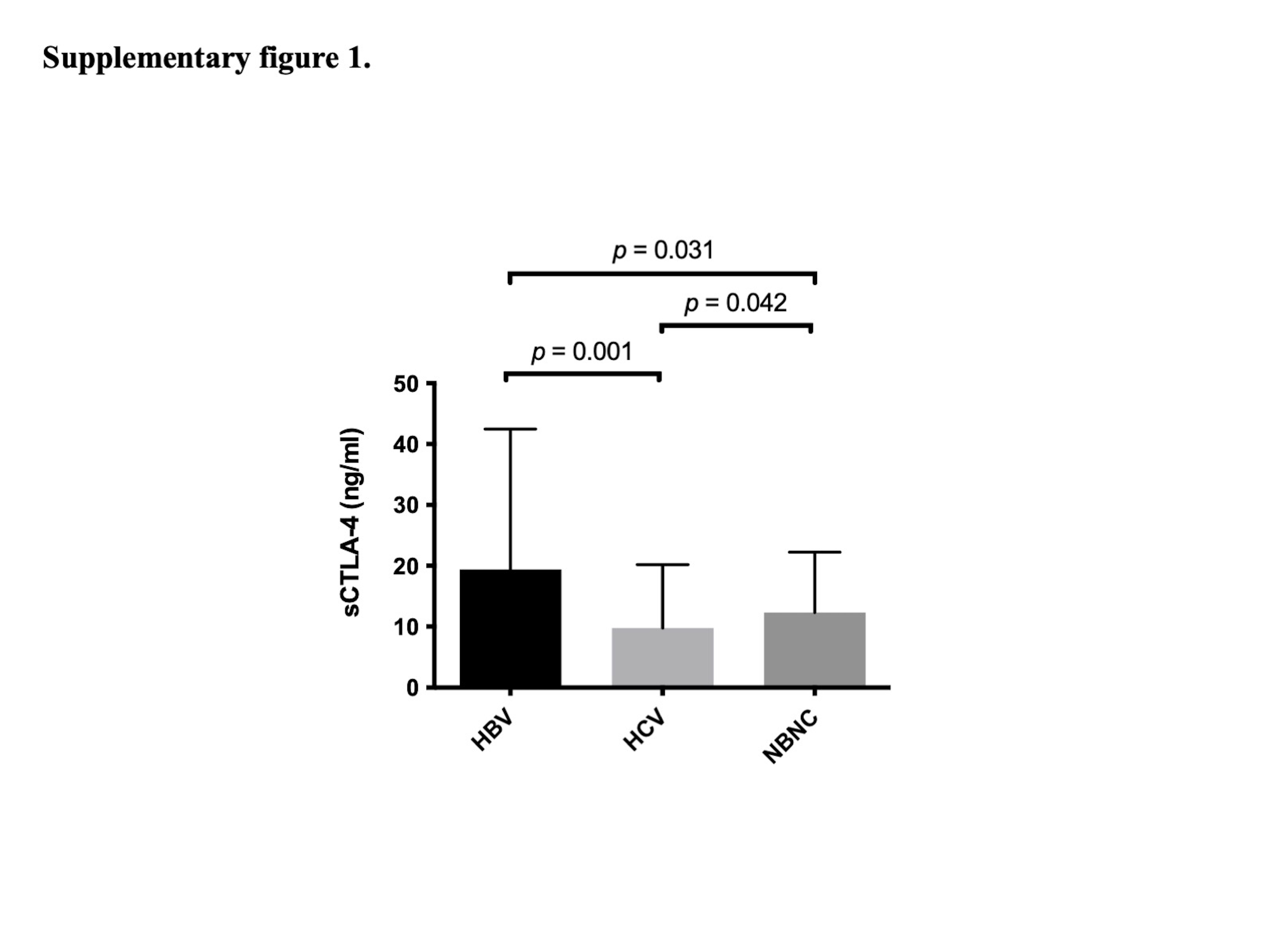

Supplement: Supplementary file 1 — Figure S1 [file CAM4-11-3786-s004.jpeg]

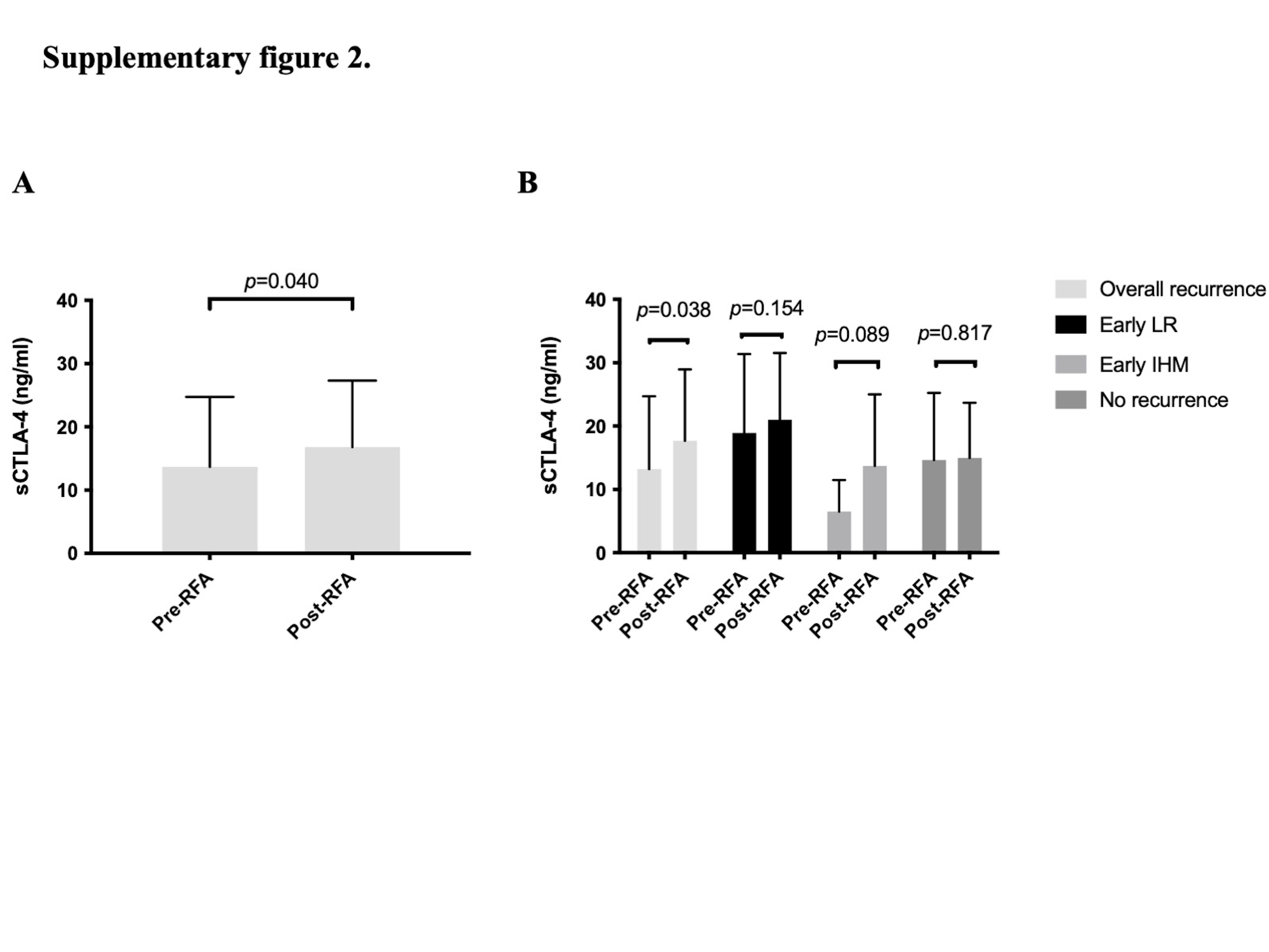

Supplement: Supplementary file 2 — Figure S2 [file CAM4-11-3786-s002.jpeg]

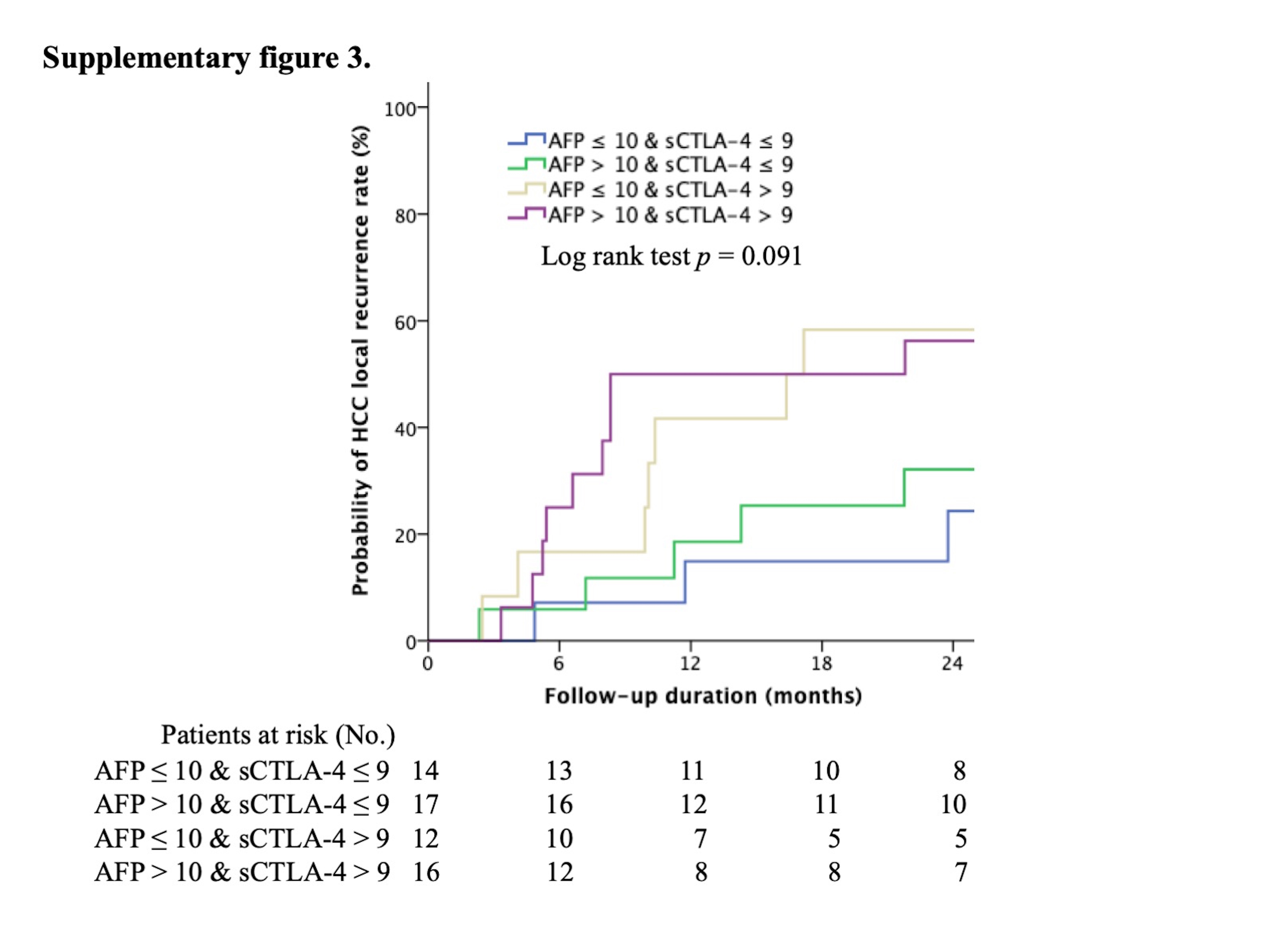

Supplement: Supplementary file 3 — Figure S3 [file CAM4-11-3786-s001.jpeg]

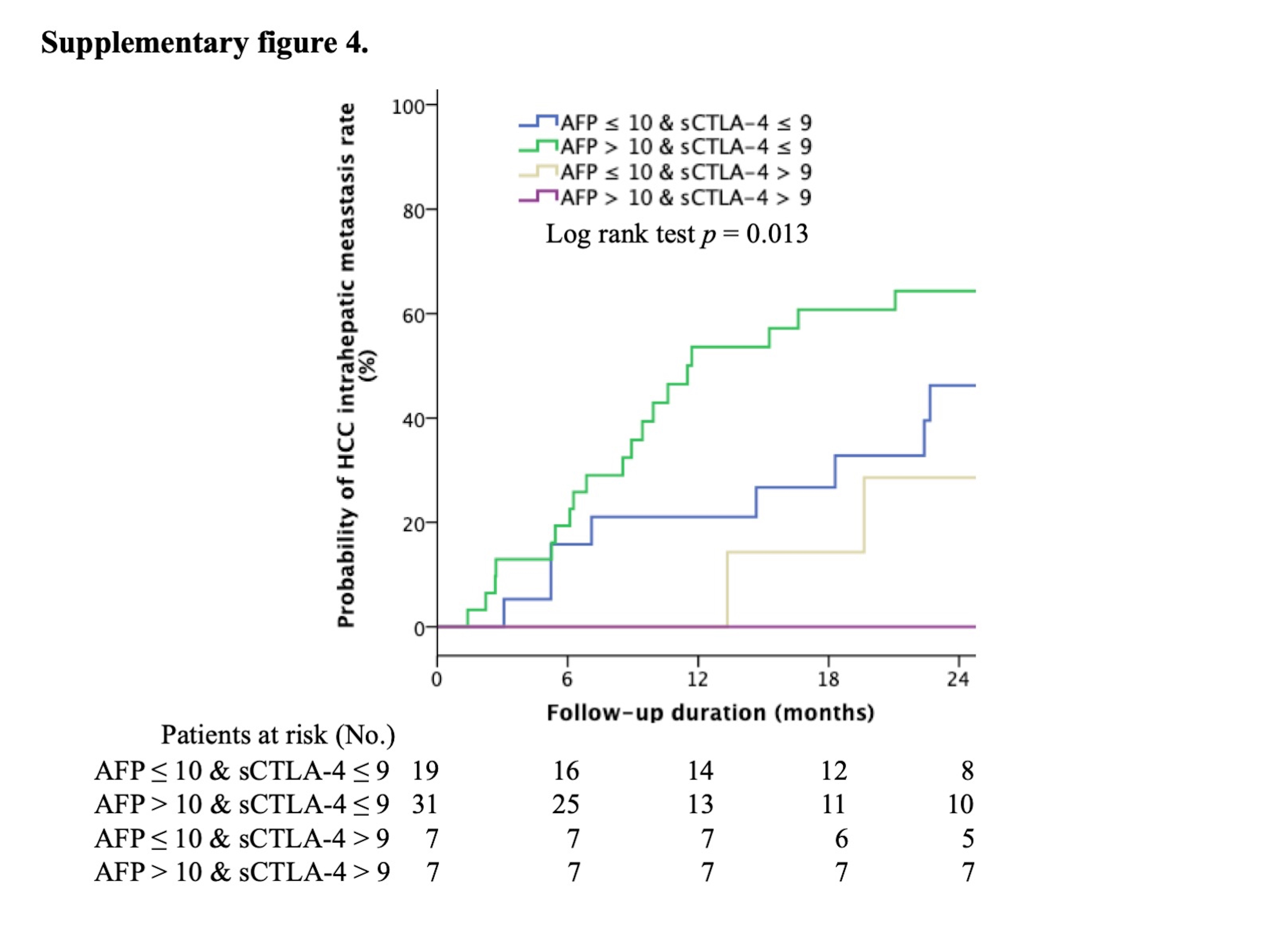

Supplement: Supplementary file 4 — Figure S4 [file CAM4-11-3786-s005.jpeg]
