# Supplementary material for: Soluble form of CTLA‐4 is a good predictor for tumor recurrence after radiofrequency ablation in hepatocellular carcinoma patients
Source: Cancer Med. 2022 Apr 18;11(20):3786–95. doi: 10.1002/cam4.4760 (PMC9582685; doi:10.1002/cam4.4760)
Supplement: Supplementary file 5 — Table S1 [file CAM4-11-3786-s003.docx]

**Supplementary Table 1. Correlation Between Serum sCTLA-4 and clinical parameters**

| **Clinical parameters** | **Correlation coefficient** | **P-value** |
| --- | --- | --- |
| Age (years) | 0.225 | 0.035 |
| Gender | 0.003 | 0.994 |
| CTP class | 0.249 | 0.014 |
| TNM stage | 0.304 | 0.027 |
| Antiviral therapy | -0.118 | 0.070 |
| ALT (U/L) | 0.093 | 0.088 |
| Total bilirubin (mg/dL) | 0.015 | 0.893 |
| Albumin (g/dL) | 0.089 | 0.420 |
| Platelet (1000/μL) | 0.103 | 0.339 |
| Target lesion size (cm) | 0.125 | 0.045 |
| AFP (ng/mL) | 0.047 | 0.664 |
| NLR | 0.274 | 0.011 |

Abbreviations: AFP, alpha-fetoprotein; ALT, alanine aminotransferase; CTP, Child-Turcotte-Pugh; NLR, neutrophil-to-lymphocyte ratio; sCTLA-4, soluble form of cytotoxic-T-lymphocyte-antigen-4
