# Supplementary material for: Soluble form of CTLA‐4 is a good predictor for tumor recurrence after radiofrequency ablation in hepatocellular carcinoma patients
Source: Cancer Med. 2022 Apr 18;11(20):3786–95. doi: 10.1002/cam4.4760 (PMC9582685; doi:10.1002/cam4.4760)
Supplement: Supplementary file 6 — Table S2 [file CAM4-11-3786-s006.docx]

**Supplementary Table 2. Comparisons of baseline features of patients with and without early tumor recurrence**

| **Variables** | **With recurrence (N=53)** | **Without recurrence (N=35)** | **P value** |
| --- | --- | --- | --- |
| Age (years) | 67.7 (IQR 62.1-75.1) | 66.9 (IQR 61.1-75.0) | 0.692 |
| Gender (male, %) | 31 (58.5) | 16 (45.7) | 0.240 |
| TNM stage I/II, n (%) | 33/20 (62.3/37.7) | 28/7 (80.0/20.0) | 0.047 |
| Antiviral therapy, n (%) | 9 (17.0) | 19 (54.3) | < 0.001 |
| CTP class A/B, n (%) | 45/8 (84.9/15.1) | 32/3 (91.4/8.6) | 0.365 |
| NLR | 2.0 ± 1.6 | 1.9 ± 1.2 | 0.857 |
| Total bilirubin (mg/dL) | 0.8 (IQR 0.6-1.3) | 0.7 (IQR 0.5-1.0) | 0.262 |
| ALT (U/L) | 38 (IQR 25-66) | 42 (IQR 25-69) | 0.778 |
| Albumin (g/dL) | 3.69 (IQR 3.36-4.13) | 3.86 (IQR 3.56-4.21) | 0.274 |
| AFP (ng/mL) | 17 (IQR 5-115) | 16 (IQR 5-66) | 0.517 |
| Platelet (x 1000/μL) | 104 (IQR 64-163) | 122 (IQR 85-164) | 0.484 |
| sCTLA-4 (ng/mL) | 10.4 ± 10.9 | 8.7 ± 9.6 | 0.520 |
| Target lesion size (cm) | 2.1 (IQR 1.6-2.9) | 1.9 (IQR 1.5-2.5) | 0.648 |
| Tumor number 1/2, n (%) | 33/20 (62.3/37.7) | 28/7 (80.0/20.0) | 0.077 |
| Mortality, n (%) | 16 (30.2) | 7 (20.0) | 0.287 |
| Follow up duration (months) | 42.0 (IQR 21.2-54.2) | 52.1 (IQR 32.0-63.6) | 0.088 |

Abbreviations: AFP, alpha-fetoprotein; ALT, alanine aminotransferase; CTP, Child-Turcotte-Pugh; HCC, hepatocellular carcinoma; IHM, intrahepatic distant metastasis; LR, local recurrence; NLR, neutrophil-to-lymphocyte ratio; sCTLA-4, soluble form of cytotoxic-T-lymphocyte-antigen-4
